# Evidence-Based Toxicology—Hypothesis Testing in Randomized Clinical Trials: Part I—Superiority

**DOI:** 10.1007/s13181-025-01109-1

**Published:** 2026-01-13

**Authors:** Joshua Trebach, Ali Graebner, Mark K. Su

**Affiliations:** 1 Department of Emergency Medicine, Division of Medical Toxicology, University of Iowa Hospitals and Clinics, Iowa City, IA USA; 2 Iowa Poison Control Center, Sioux City, IA USA; 3 New York-Presbyterian Brooklyn Methodist Hospital, Brooklyn, NY USA; 4 Ronald O. Perelman Department of Emergency Medicine, Division of Medical Toxicology, NYU Grossman School of Medicine, New York, NY USA; 5 Department of Health and Mental Hygiene, New York City Poison Control Center, New York, NY USA

**Keywords:** Superiority trial, Clinical trial, Randomized clinical trial

“The only way to test a hypothesis is to look for all the information that disagrees with it.”

-Karl Popper

The word “hypothesis” originates from Greek *hypo* (“under”) and *thesis* (“placing”), and its ancient usage was as “a summary of the plot of a classical drama.” Here we will delve into the modern scientific dramas that are known as randomized controlled trials and discuss the role of the hypothesis.

In modern day research, the hypothesis has evolved to be more than just a “summary” or an assumption of what a researcher thinks will happen. Specifically, a hypothesis is an assumption that is being tested. The hypothesis sets the stage for the rest of the study in terms of both the study design and data analysis. There are three major types of study designs that frame the hypothesis a researcher can choose–superiority, equivalence, and non-inferiority [1]. It should be noted that although the terms “study” and “trial” may seem interchangeable, they are not the same. A “study” refers to an investigation performed to obtain knowledge or understanding of a topic. For example, if investigators want to determine if there’s a difference in outcomes for patients with alcohol withdrawal syndrome treated with lorazepam vs diazepam, this would be classified as a study. In contrast, a “trial” is a specific type of study where the effects of an intervention or treatment are evaluated compared to the current conventional (i.e., standard) treatment. One type of trial, the randomized controlled trial, can be further categorized based on the nature of its hypothesis, be it a superiority trial, equivalence trial, or non-inferiority trial. Essentially, while all trials are studies, all studies are not trials.

There are also two types of hypotheses—a research hypothesis and a statistical hypothesis. A research hypothesis is a specific prediction made based on theoretical ideas/concepts: “Treatment A reduces hypertension in adults.” A statistical hypothesis, on the other hand, is a testable prediction with formal parameters: “Treatment A reduces hypertension by 20% more than Treatment B.” While both the research and statistical hypothesis can be identical in some situations, other times there are notable differences.

Before generating a hypothesis and performing clinical research, researchers must start by considering the general study design. For example, if investigators want to perform a randomized controlled trial, they must consider the intervention being tested (e.g., a new antidote), the primary outcome of interest (e.g., survival), and if the intervention can be ethically randomized to a treatment and control group [2]. It is also imperative that the researchers determine whether *equipoise* exists for the study hypothesis [3]. Equipoise refers to a state of general uncertainty in which the researchers are not sure which treatment arm (intervention(s) or control) is better or worse. Furthermore, equipoise must always be considered throughout the entire study and if one treatment arm is clearly demonstrated to be superior (or harmful) at an interim analysis, the research study must be terminated early.

When creating a statistical hypothesis, researchers must ask themselves “What do we think is going to happen?” Clinical significance is a predetermined value (often based on existing data or previous clinical experience) that the researchers deem would make their findings *clinically significant*. This is different from *statistical significance*, a purely mathematical calculation which results in a p-value that can inform whether or not to reject null hypothesis [4, 5]. For example, in a trial of a new antibiotic, researchers may determine beforehand that the new antibiotic will be deemed clinically significant if it decreases infection duration by 72 h. However, if the trial finds that the new antibiotic decreases infection duration by 1 h, while this could be statistically significant, it is unlikely to be clinically significant. It should also be noted that the study (or research) hypothesis and statistical hypothesis are distinctly different terms. The study hypothesis is the proposed “answer” to the research question or predicted relationship between the study variables; the statistical hypothesis (i.e., null and alternative hypotheses) is a more specific statement about a population parameter that can be tested using statistical methods. Furthermore, to test certain statistical hypotheses, it should be noted that choice of effect size can be selected based on prior published studies (including animal studies). In some circumstances, when there is a lack of published relevant literature, simulation studies (computer-based experiments that model real life) could be performed to determine potentially clinically significant effect sizes. Effect sizes, significance, and sample size calculations should all be done by the researchers prior to beginning the trial. Researchers should also keep in mind that randomized controlled trials with smaller effect sizes will likely require larger samples, which can be an increased burden on resources/funding.

There are three common trial types with different approaches to hypothesis testing—superiority, equivalence, and non-inferiority. Each of these trials involves a null hypothesis (H_0_) and alternative hypothesis (H_a_). The null hypothesis, by definition, is that there is no difference between the two treatments being tested. In contrast, the alternative hypothesis is the hypothesis if the null hypothesis is rejected and it typically reflects the research question being studied.

In Part I of this three part series, we will start with a discussion of the most common and familiar type of trial–the superiority trial.

## Superiority

A superiority trial tests a hypothesis where a new treatment is better than what currently exists or the “gold standard.” As an example of a hypothetical superiority toxicology trial, if a researcher wants to test a hypothetical new drug (“WAC”) compared to NAC for the treatment of acetaminophen toxicity, the null hypothesis (H_0_) and alternative hypothesis (H_a_) would be as follows:

- H_0_ = WAC is *not* superior to NAC in treating acetaminophen toxicity.
- H_a_ = WAC is superior to NAC in treating acetaminophen toxicity.

Because this is a superiority trial, the alternative hypothesis here is that the new treatment is *superior* to the standard treatment. It is worth noting that in randomized controlled trials, “standard” treatment is typically the current gold standard treatment at the time of the study. It should also be noted that in some cases where a currently accepted “gold standard” treatment is lacking, a placebo or “usual care” can be the comparator.

A common misconception regarding superiority studies is assuming that when the null hypothesis is not rejected that the two drugs (WAC and NAC) are *equivalent*. However, this is not necessarily true since a superiority study only addresses a binary yes/no question: Is the new drug (WAC) superior to the currently existing gold standard treatment (NAC)?

Let us say we want to conduct a superiority study comparing WAC and NAC. Before the study begins, we determine our effect size (δ); superiority will be demonstrated if WAC performs 20% better than NAC based on a specific patient outcome (e.g., fulminant hepatic failure). In this case, the effect size chosen is “20%.” To determine the effect size, in general, the investigators typically use prior literature to determine the baseline prevalence of the outcome of interest and then choose how much better the new treatment should be for their results to be clinically significant. The investigators’ choice of effect size is a critical decision that is made a priori to trial initiation. If the investigators choose a small effect size, this may make it easier to find statistical significance, but this difference may not be clinically meaningful, and a larger sample size is usually needed. In contrast, if the investigators choose an effect size that is too large, fewer patients may be needed but the trial may not be able to statistically demonstrate this significance even if the difference is clinically meaningful (i.e., increasing the risk of a type II error). The chosen effect size therefore has to “make sense” and be something that most readers would consider clinically meaningful. Furthermore, when combining effect size with p-values, readers of the research are better able to make assessments about the effect of one intervention compared to another beyond simply stating that a statistically significant difference exists (i.e., the null hypothesis has been rejected). After the trial has been completed, we can then assess (or test) the difference in outcome between our new drug WAC and our standard drug NAC (represented on the figure below as a blue circle) and the corresponding confidence intervals.

**Fig. 1:**
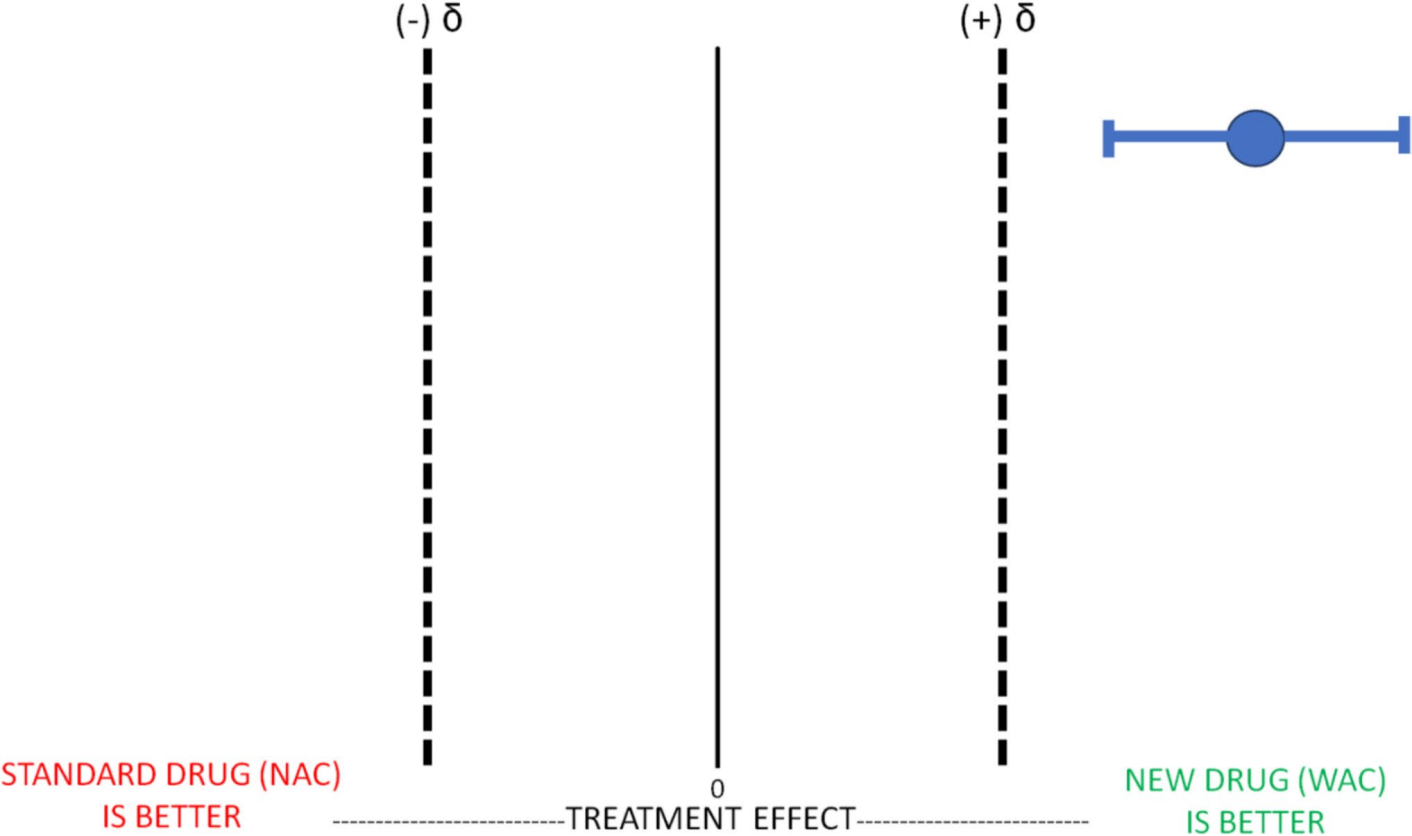
Superiority Trial: WAC Superior to NAC. Legend: In a Superiority Trial, for the new experimental drug WAC to be considered superior to the standard treatment NAC, the mean treatment effect and it’s CI (typically 95%) must be above the a priori estimated effect size difference (δ). In this example, WAC demonstrates superiority to NAC because it meets this definition

This figure illustrates that WAC is superior to NAC as our mean value and our confidence intervals are above our predetermined effect size (Fig. 1).

**Fig. 2:**
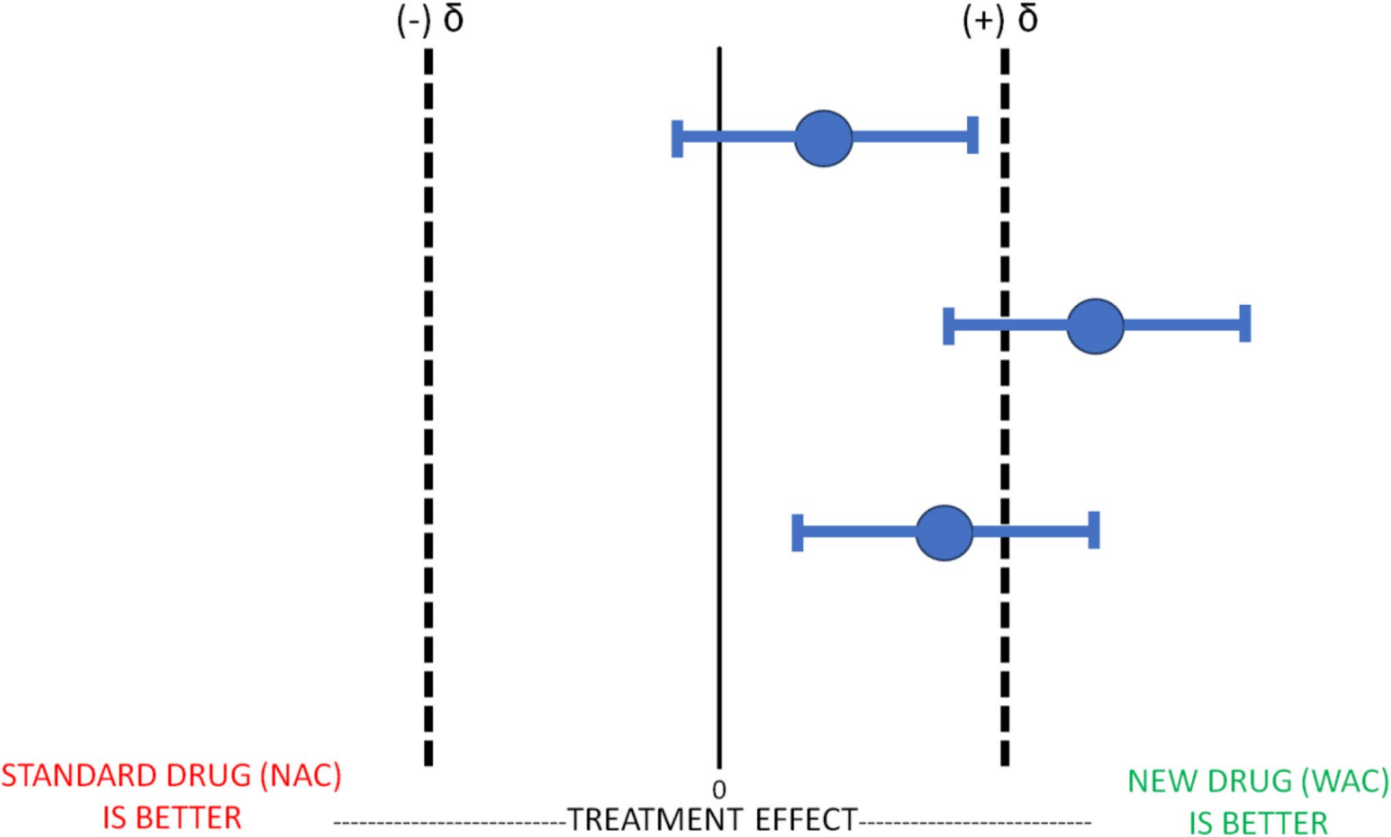
Superiority Trial: WAC Not Superior to NAC. Legend: This figure illustrates the results of three separate superiority trials comparing the experimental drug WAC to the standard therapy NAC. In Superiority Trials, the new experimental drug WAC cannot be considered superior to the standard treatment NAC in any of these three trials because the mean treatment effects and their CIs are either overlapping with a priori estimated effect size difference (δ) or less than the estimated effect size difference. All three trials would therefore be considered “not superior”

This figure below (Fig. 2) represents multiple iterations of the trial where WAC is not superior to NAC since the mean value and/or their corresponding confidence intervals do not exceed (i.e., overlap) our predetermined effect size. It should be noted again that the lack of finding superiority of WAC to NAC should not be interpreted as WAC is worse than NAC or that WAC is equivalent to NAC.

Of the three trial types (superiority, equivalence and non-inferiority), superiority trials are the specific trial design to use to determine if a new interventions is better than current standard treatment. If a superiority trial demonstrates superiority of a new intervention, the results could be potentially “practice-changing.” However, even if a trial does not demonstrate superiority, the results may still be meaningful because they show that the new intervention may have little or no value and should no longer be considered as a potential treatment. Moreover, if a superiority trial fails to demonstrate superiority, there can be some other additional important implications or considerations [6]:

1. The dosing of the new intervention may have been incorrect (i.e., too low or too high)
2. The technical skill of those providing the intervention (e.g., surgery skills) may have been suboptimal
3. The sample size was too small to detect effects that might have been smaller than the originally prespecified power calculations
4. There was poor adherence with the assigned treatment group
5. Concomitant interventions given simultaneously could have diminished the effect of the intervention
6. There was inadequate sensitivity of outcome measurements to determine the primary outcome
7. Unmeasured or measured confounders which may suppress the association between treatment and outcome
8. Patients that were not followed until the primary outcome was reached (i.e., attrition bias)

Since RCTs are considered to be performed under “ideal” conditions, repeated studies and/or registries can help determine follow-up effects of the treatment in different populations to determine whether there is reproducibility of the trial results.

Superiority trials, in contrast to non-inferiority trials and equivalence trials, are often the most straightforward of the hypothesis testing RCTs and are fairly robust especially with regards to missing data. However, superiority trials are not always feasible due to available data, resources, costs, sample size requirements, etc. In these cases, researchers can therefore look to non-inferiority trials and/or equivalence trials as alternative study designs.

## References

[1] Christensen E. Methodology of superiority vs. equivalence trials and non-inferiority trials. J Hepatol. 2007;46(5):947–54. 17412447 10.1016/j.jhep.2007.02.015

[2] Sugarman J, Stolbach A. Ethics and medical toxicology research. J Med Toxicol. 2017;13:255–8. 28540608 10.1007/s13181-017-0618-4 PMC5570727

[3] Freedman B. Equipoise and the ethics of clinical research. N Engl J Med. 1987;317(3):141–5. 10.1056/NEJM198707163170304. 3600702 10.1056/NEJM198707163170304

[4] Willigenburg NW, Poolman RW. The difference between statistical significance and clinical relevance. The case of minimal important change, non-inferiority trials, and smallest worthwhile effect. Injury. 2023;54(Suppl 5):110764. 37923502 10.1016/j.injury.2023.04.051

[5] Ferrill MJ, Brown DA, Kyle JA. Clinical versus statistical significance: interpreting P values and confidence intervals related to measures of association to guide decision making. J Pharm Pract. 2010;23(4):344–51. 21507834 10.1177/0897190009358774

[6] Friedman LM, et al. *Fundamentals of Clinical Trials*, Springer International Publishing AG, 2015. *ProQuest Ebook Central*. Accessed at https://ebookcentral-proquest-com.ezproxy.med.nyu.edu/lib/nyulibrary-ebooks/detail.action?docID=5586421.

